# Supplementary material for: Walnut (J. regia) Agro-Residues as a Rich Source of Phenolic Compounds
Source: Biology (Basel). 2021 Jun 15;10(6):535. doi: 10.3390/biology10060535 (PMC8232793; doi:10.3390/biology10060535)

Supplementary materials

Figure 1. MS[1] spectra of hydrojuglone β-D-glucopyranoside (naphthoquinone) in a negative ion mode.

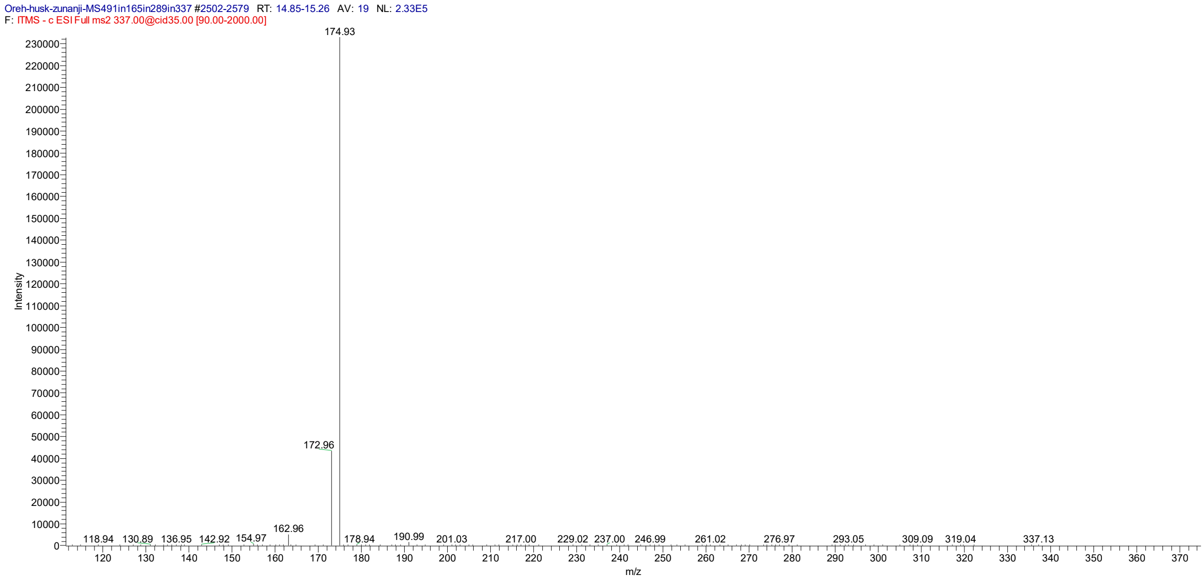

Figure 2. MS[2] spectra of hydrojuglone β-D-glucopyranoside (naphthoquinone) in a negative ion mode.

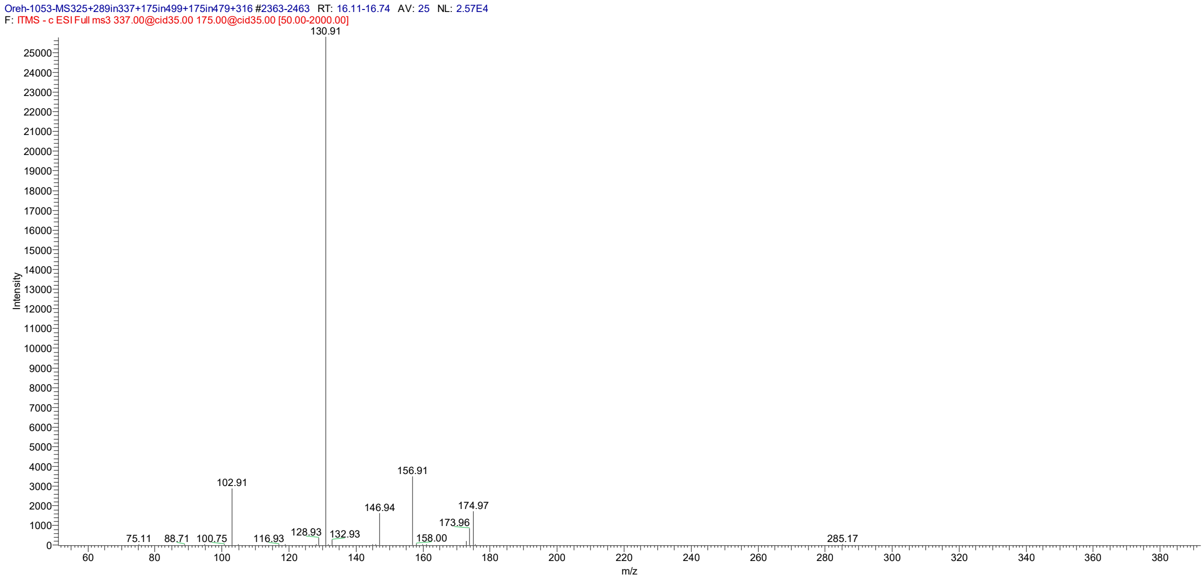

Figure 3. MS[1] spectra of gallic acid derivative 5 (hydroxybenzoic acid) in a negative ion mode.

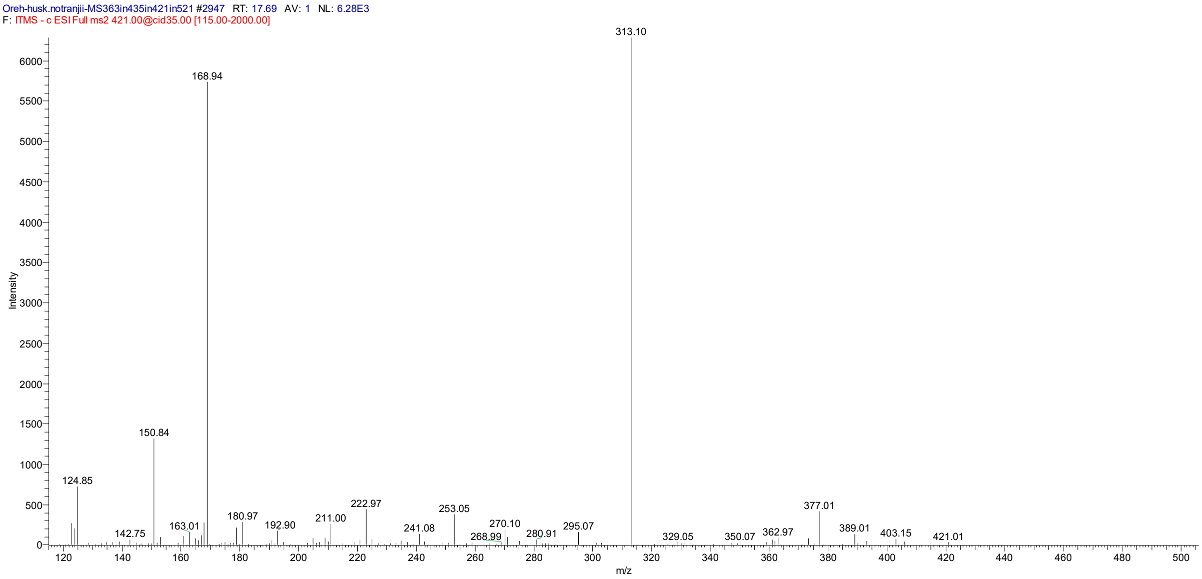

Figure 4. MS[1] spectra of (epi)catechin derivative 5 (flavanol) in a negative ion mode.

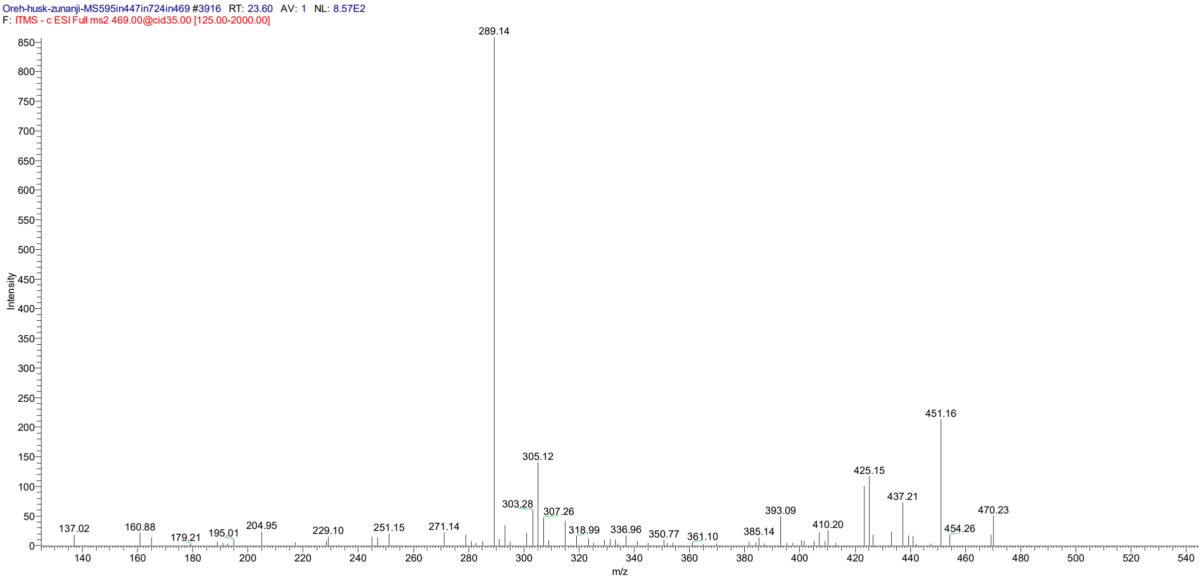

Figure 5. MS^2 spectra of (epi)catechin derivative 5 (flavanol) in a negative ion mode.

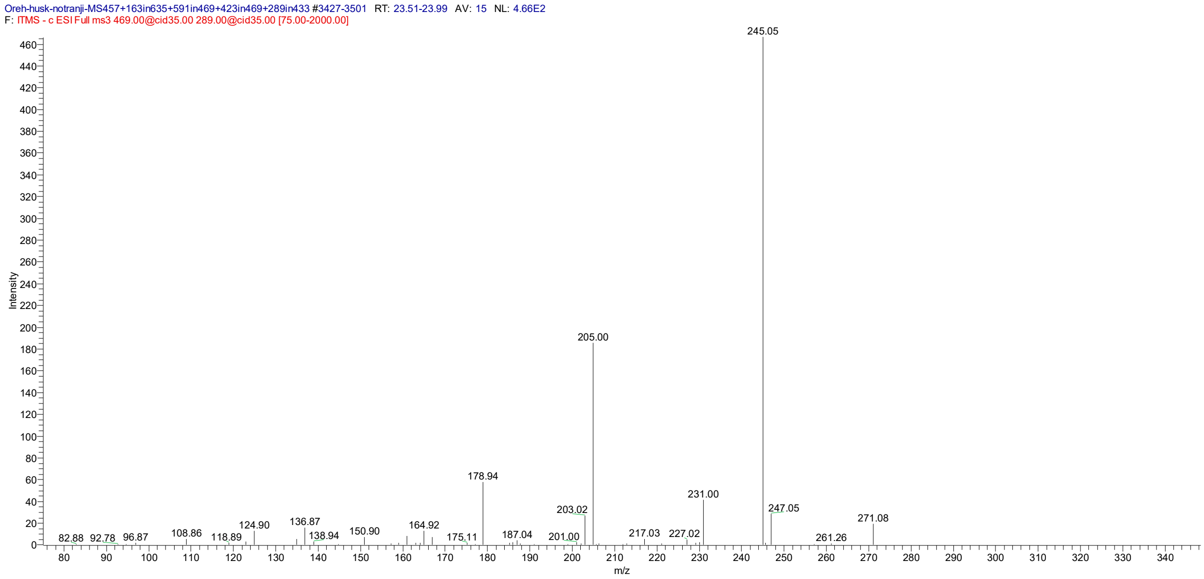

Figure 6. MS^1 spectra of santin (flavone) in a negative ion mode.

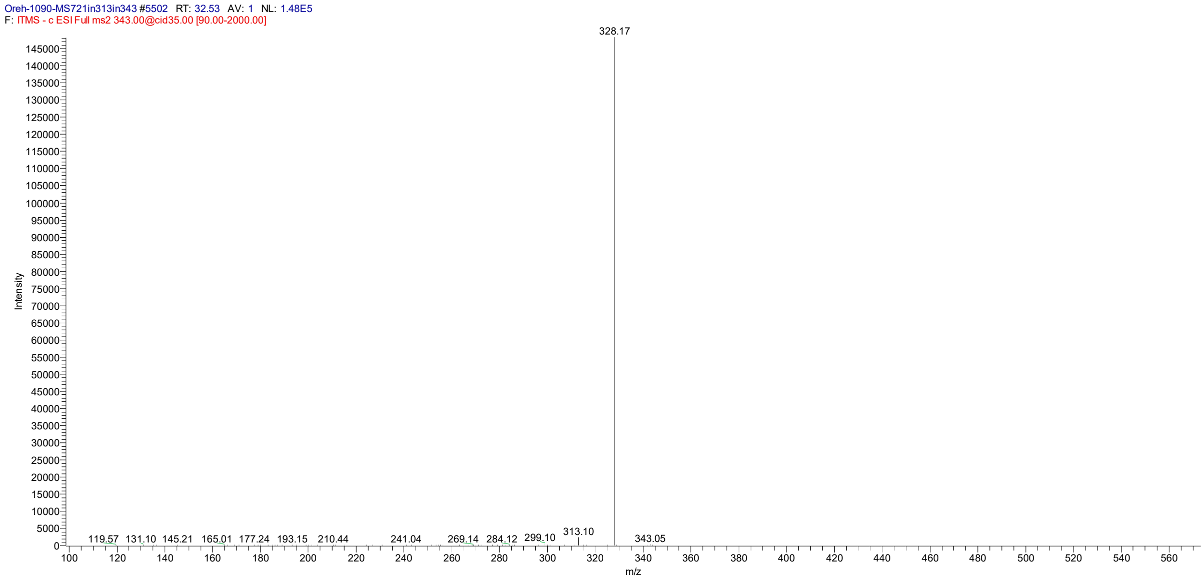

Figure 7. MS^2 spectra of santin (flavone) in a negative ion mode.

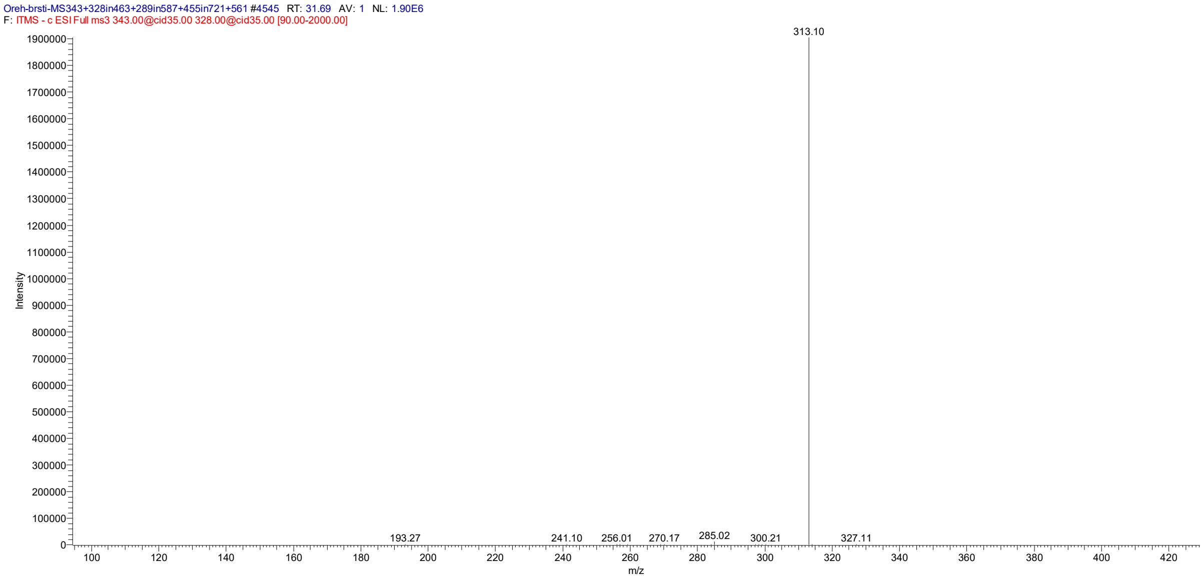

Figure 8. MS^1 spectra of quercetin-rhamnoside (flavonol) in a negative ion mode.

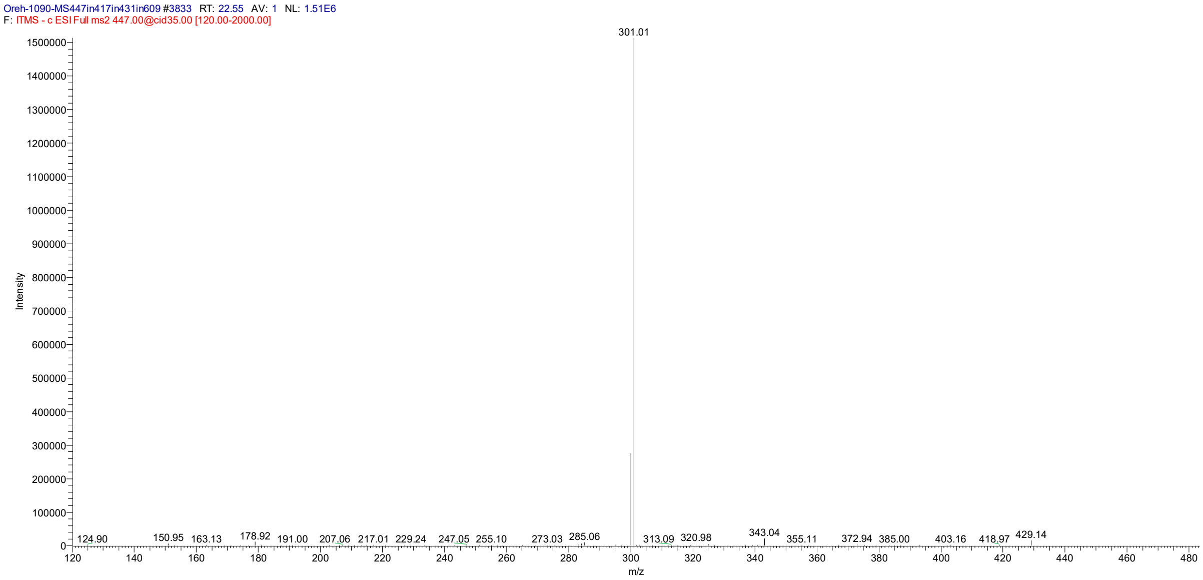

Figure 9. MS^2 spectra of quercetin-rhamnoside (flavonol) in a negative ion mode.

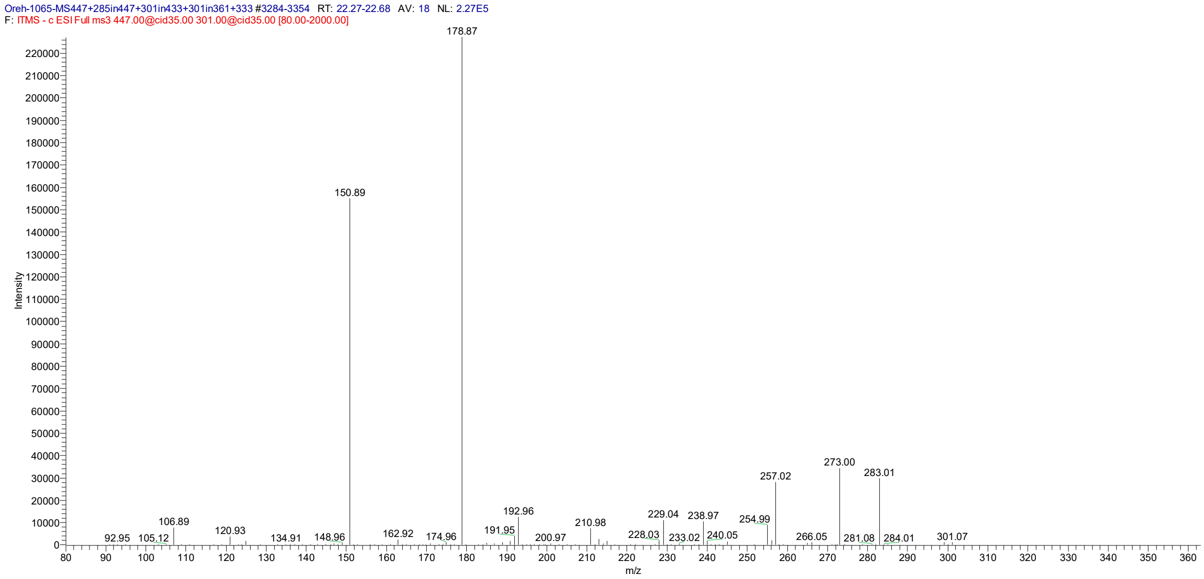

Figure 10. MS^1 spectra of *p*-coumaric acid derivative 4 (hydroxycinnamic acid) in a negative ion mode.

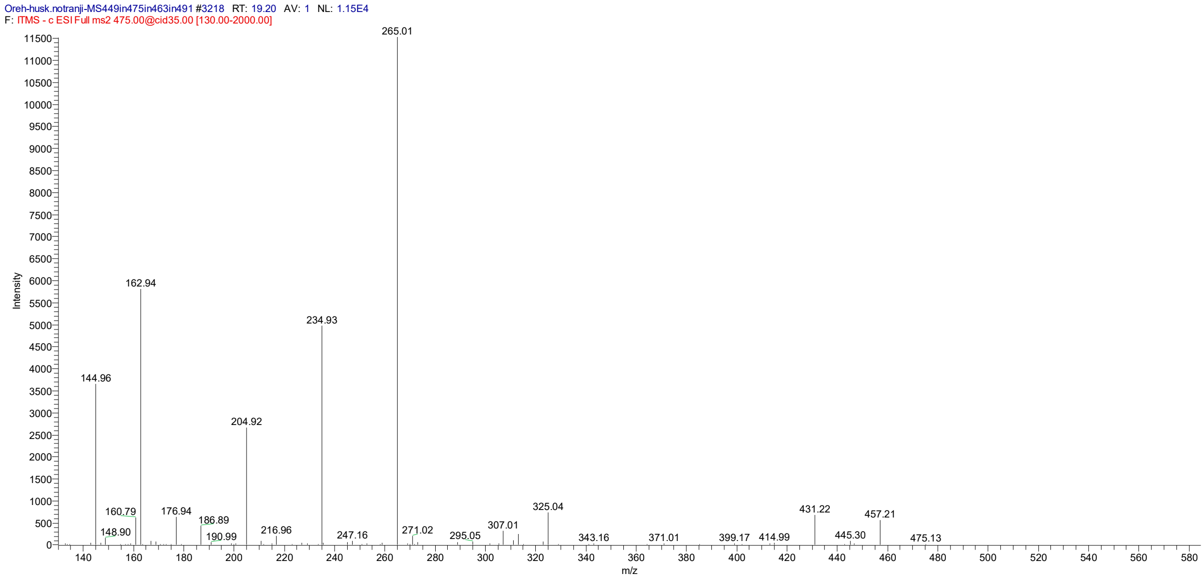

Figure 11. MS^2 spectra of *p*-coumaric acid derivative 4 (hydroxycinnamic acid) in a negative ion mode.

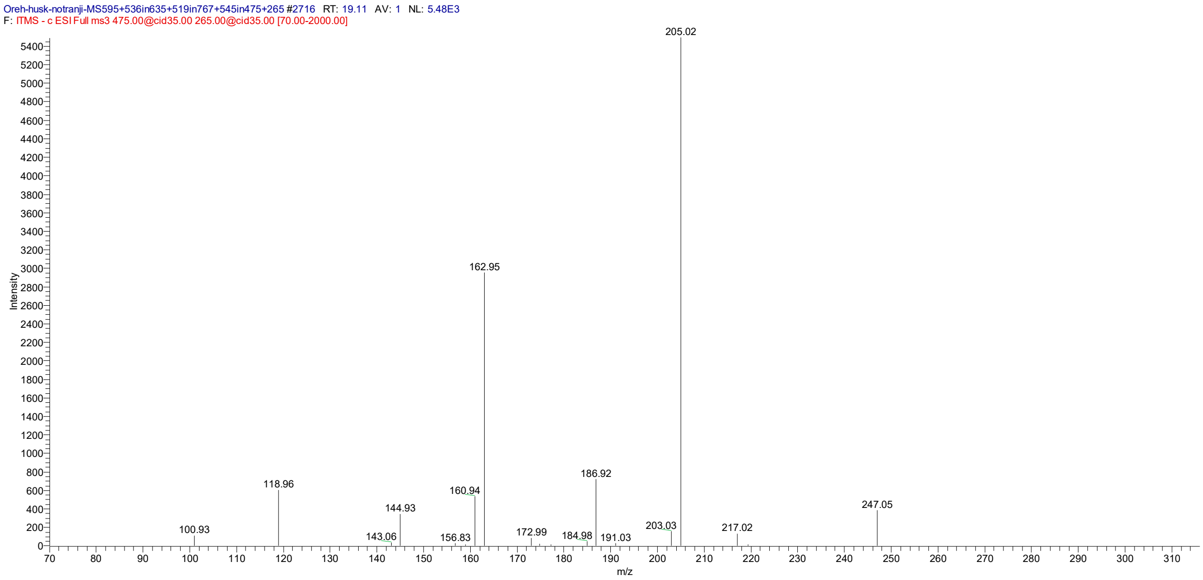

Supplement: Supplementary file 1 [file biology-10-00535-s001.zip › biology-1252153-supplementary.pdf]
